# Financial Incentives and Maternal Health: Where Do We Go from Here?

**Published:** 2013-12

**Authors:** Lindsay Morgan, Mary Ellen Stanton, Elizabeth S. Higgs, Robert L. Balster, Ben W. Bellows, Neal Brandes, Alison B. Comfort, Rena Eichler, Amanda Glassman, Laurel E. Hatt, Claudia M. Conlon, Marge Koblinsky

**Affiliations:** ^1^Broad Branch Associates, Washington, DC, USA; ^2^US Agency for International Development, Washington, DC, USA; ^3^Division of Clinical Research, National Institute of Allergy and Infectious Diseases, Bethesda, MD, USA; ^4^Virginia Commonwealth University, Richmond, VA, USA; ^5^Population Council, Nairobi, Kenya; ^6^Abt Associates Inc., Bethesda, MD, USA; ^7^Center for Global Development, Washington, DC, USA

**Keywords:** Healthcare-seeking behaviour, Health services research, Maternal health services, economics/utilization, Motivation, Newborn health services, Pregnancy, Programme evaluation

## Abstract

Health financing strategies that incorporate financial incentives are being applied in many low- and middle-income countries, and improving maternal and neonatal health is often a central goal. As yet, there have been few reviews of such programmes and their impact on maternal health. The US Government Evidence Summit on Enhancing Provision and use of Maternal Health Services through Financial Incentives was convened on 24-25 April 2012 to address this gap. This article, the final in a series assessing the effects of financial incentives—performance-based incentives (PBIs), insurance, user fee exemption programmes, conditional cash transfers, and vouchers—summarizes the evidence and discusses issues of context, programme design and implementation, cost-effectiveness, and sustainability. We suggest key areas to consider when designing and implementing financial incentive programmes for enhancing maternal health and highlight gaps in evidence that could benefit from additional research. Although the methodological rigor of studies varies, the evidence, overall, suggests that financial incentives can enhance demand for and improve the supply of maternal health services. Definitive evidence demonstrating a link between incentives and improved health outcomes is lacking; however, the evidence suggests that financial incentives can increase the quantity and quality of maternal health services and address health systems and financial barriers that prevent women from accessing and providers from delivering quality, lifesaving maternal healthcare.

## INTRODUCTION

All over the world, prospects for women and their babies are improving. Between 1990 and 2010, maternal deaths declined by nearly 50% worldwide (http://www.who.int/mediacentre/factsheets/fs348/en/). The use of effective maternal health interventions, such as uterotonics to prevent excess bleeding and magnesium sulphate to treat severe pre-eclampsia and eclampsia, is increasing. Option B+ for prevention and treatment of HIV/AIDS in pregnant women is being initiated (Under Option B+, all pregnant women living with HIV are offered lifelong ART, irrespective of their CD4 count). Intermittent Preventive Treatment (IPT) in pregnancy and long-lasting insecticide-treated bednets are proving effective in reducing the risk of malaria infection among pregnant women, with benefits to both mothers and their children. Yet, despite significant political and financial commitments and technological advances, underutilization of services and poor quality provision persist. As a result, about 287,000 women continue to die each year from complications relating to pregnancy and childbirth—about one in every two minutes (1). The vast majority of these women live in poor countries and the vast majority of these deaths are preventable.

### The importance of (dis)incentives

Transforming effective interventions into improved health outcomes requires tackling the disincentives patients and providers face in taking actions that lead to better health. Better health requires that individuals demand and are able to access services, that providers are motivated to deliver quality care (and have the inputs needed to do so), and that managers at all levels are encouraged to address systemic barriers to achieving health goals. The choices that both patients and providers make are influenced by incentives in the health systems that enable or constrain them and drive their behaviour.

Many disincentives exist that may prevent a woman and her family from seeking and reaching care due to inadequate knowledge, low levels of perceived need, social norms and taboos, transportation costs, opportunity costs of time-off from work, and the logistical costs associated with childcare. Furthermore, user fees at the point of service may lead households to prioritize urgent curative care services and neglect preventive care (2,3).

On the supply side, lack of supervision and support, inadequate numbers of providers, along with low, fixed salaries that do not vary based on performance, may not spur health providers to creatively solve problems and can lead to low productivity, absenteeism, clinical care of poor quality, lack of innovation, and even disrespectful care. Reimbursement for expenses can encourage providers to devote time and energy to tracking and justifying inputs rather than to expanding coverage, promoting preventive services, or solving systemic problems, even when they have the intrinsic motivation to do so.

### Financial incentives

Health financing strategies that incorporate financial incentives aim to address these issues by providing a direct link between money spent and results generated. On the supply side, performance-based incentives (PBIs) aim to spur providers to focus on improvements in the quantity and quality of services by paying incentives only when such results have been delivered and verified. Demand-side programmes also incentivize results—the utilization of services. Incentives, thus, aim to minimize financial barriers to seeking and accessing services while also holding providers accountable for results.

Although the use of financial incentives for maternal health is growing, clarity on the state of the evidence supporting the effectiveness and sustainability of these interventions has been lacking. Yet, governments and donors need evidence to guide policy and practice. With this in mind, the US Government Evidence Summit was held on 24-25 April 2014 to review the evidence on financial incentives and provide recommendations for policy, practice, and research (Detailed results of the evidence review process are provided in other articles in this Supplement of the Journal). The Summit focused almost exclusively on evidence generated from programmes in low- and middle-income countries (LMICs). During the Evidence Summit process, the panels of experts assembled and systematically reviewed the evidence with the aim of answering the two key questions as follows:

1. What financial incentives, if any, are linked positively or negatively to maternal and neonatal health outcomes, the provision and use of maternal health service, or to care-seeking behaviour by women?
2. What are the contextual factors that impact the effectiveness of these financial incentives?

This paper summarizes the key findings from our Evidence Summit reviews—which together synthesizes 86 studies of 60 programmes, identifies gaps in evidence that can help shape the research agenda of the future, and offers suggestions for strengthening incentive programmes to improve maternal, neonatal and broader health outcomes.

### Financial incentives reviewed in this series

The Evidence Summit covered a wide range of financial incentive instruments implemented across a range of settings (See Box). Among the many countries represented, there exist myriads of differences in economic status, size, population density, baseline health status, and political and social context, and within each financial incentive category, there are also tremendous variations. Among programmes that offer incentives to providers, we reviewed performance-based contracting of non-governmental organizations (NGOs), programmes that give incentives to public-sector facilities, incentives that are implemented as part of a social insurance reform, and safe-delivery programmes that provide incentives both to providers and patients (4). User fee exemption programmes vary based on which service fees are exempted, if fees are abolished for certain groups or entire populations, and whether and how providers are compensated for the loss of revenue from the fees (3). Voucher programmes vary based on the types of facilities eligible to participate, which services are covered, and how these are managed (whether by a social franchise, NGO, or private firm), among other variables (5). We also review a variety of insurance schemes, namely social insurance, public health insurance, community-based insurance, and private coverage schemes (6).

Although incentive programmes are often categorized as focusing either on the ‘demand’ for or ‘supply’ of healthcare, many programmes have components that target both patients and providers. Insurance and voucher programmes, for instance, aim to generate demand but they also provide incentives to providers in the form of fees paid for seeing insured/voucher patients. Some conditional cash transfer (CCT) programmes include components that support the supply side, and some PBI programmes also offer incentives to patients.

Our review spans the peer-reviewed and grey literature (7). There is significant variation in the literature in terms of the methods used for evaluating the impact of programmes, ranging from simple before-after comparisons using baseline and endline data, to various econometric methods that attempt to control for potential biases and confounding factors.

About half of the studies in Comfort *et al.* (the insurance review) used econometric analyses; none used a randomized control trial design. The studies reviewed by Hatt *et al.* (user fees) use mostly descriptive statistics and qualitative and case study research approaches. Only a few studies use pre/post designs, with no controls; and only one examines changes over time with controls. The CCT studies are the most rigorous set of studies comprising well-designed impact evaluations with experimental or quasi-experimental designs and output measures that are relatively comparable and consistent across different studies. Among the small number of PBI studies, only one shows results from a large-scale impact evaluation with randomly-assigned intervention and control facilities. Among the 15 studies of maternal health voucher programmes, only four used a before-after with controls or quasi-experimental design. The remainder used cross-sectional or before-after designs.

**Box. Types of incentive instruments reviewed**

*Conditional cash transfers* (CCT): ‘Broad’ CCT programmes make regular cash payments to poor households conditional on the use of certain health services and school attendance in order to provide a safety net to increase and smooth the consumption of the extreme poor (alleviating short-term poverty) and to increase the human capital investment of poor households (alleviating long-term poverty). ‘Narrow’ CCT programmes make one-time cash payments for the utilization of specific services (8).

*Insurance*: Insurance allows individuals to protect themselves against the financial cost of illness by pooling risks with others in the population by making small, regular payments which may be partially or fully subsidized by the government or a donor agency. Providers may receive capitation payments or submit claims for reimbursement (6).

*Performance-based incentives* (PBI): Programmes that provide incentives to healthcare providers when they achieve performance targets in the quantity and quality of care provided; or to health managers at the district, provincial, and national level, conditional on such things as timely and accurate reporting or the performance of the facilities they are responsible for (4).

*User fee exemptions*: Programmes that provide exemptions from fees charged to consumers for specific services (3).

*Vouchers*: Programmes wherein a purchaser contracts accredited health facilities and vouchers are distributed to patients entitling them to services at those facilities. The voucher is either heavily subsidized or free for the patient, and the provider is reimbursed for the cost of service provision plus a reasonable profit after service delivery has been verified (5).

In short, the body of evidence is of variable quality; on the whole, it provides indicative but not conclusive evidence of the causal impact of the incentive instruments on outcomes.

## RESULTS

### Overview of findings

#### Incentives linked with increases in service utilization

Most studies report increases in the quantity of key maternal health services utilized (Table). This finding holds across incentive instruments and geographic locations. Increases in the quantity of services are especially significant where baseline access is low and occurs in some cases in remarkably short timeframes. In some programmes, indicators for which improvements are observed are directly incentivized. In others, these are not; in these cases, improvements appear to be positive spillovers.

**Table.** Summary of the effects of incentives on quantity of services

Type of service	Type of incentive				
	PBI	Insurance	User fee exemptions	CCTs	Vouchers
	9 programmes/9 studies	19 programmes/29 studies	13 programmes/19 studies	8 programmes/14 studies	11 programmes/15 studies
ANC	- Incentivized directly in 7/9 programmes	- Most articles do not specify which services are covered by the insurance programme	- ANC part of exempted services in 5/13 programmes - Malaria care for pregnant women exempted in 2/13 programmes	- 7 programmes provided incentives for ANC	- All programmes cover ANC
	Findings: - Afghanistan: increases in ANC visits - Rwanda: no increase in number of ANC visits but increase in quality of ANC as measured by provision of tetanus toxoid vaccine - Egypt: quality of ANC care improved; unclear if quantity increased - No increase in number of ANC visits in Bangladesh, Cambodia, DRC, and Haiti - No change reported in Nepal or Philippines	Findings: - Most studies found a positive relationship between health insurance and both probability of women using any ANC and the probability of women receiving at least four ANC visits during their pregnancy	Findings: - 1 study showed that where pregnant women were exempted from fees for malaria-related services, the use of such by pregnant women increased - 1 study in South Africa documented a decrease in ANC service-use when fees for curative care were removed - Study in Afghanistan found that removing user fees for primary healthcare services did not have any effect on ANC visits - Study from Nigeria identified an increase in ANC utilization rates over the 5-year period after free ANC and maternity care was introduced	Findings: - 5 studies report positive and significant increases in the average number of beneficiaries that received at least 5 antenatal visits among beneficiaries compared to non-beneficiaries	Findings: - 6 studies of 4 programmes report increases in ANC
Delivery	- 8/9 programmes directly incentivize delivery	- Most articles do not specify which services are covered by the insurance programme	- Women exempted from delivery and/or C-section fees in 10/13 programmes	- 6 programmes provided incentives for births attended by skilled personnel, and 3 studies provided incentives for facility-based delivery	- All programmmes cover delivery - Most programmes include C-section
	Findings: - Majority of programmes show PBI associated with increases in the quantity of institutional deliveries - Only a small study in the DRC did not find an association between PBI and the number of institutional deliveries	Findings: - Mostly consistent evidence that health insurance positively correlated with facility-based delivery and skilled attendance at birth - 6 studies demonstrate a positive correlation between insurance and provision of C-sections; none proves supplier-induced demand	Findings: - User fee removal for facility-based deliveries resulted in increased facility-based delivery rates and C-section in some contexts	Findings: - 5 studies reported positive effects of CCT on whether a woman's last childbirth was attended by skilled personnel - 3 reported positive effects on whether a woman's last childbirth occurred in a hospital - 2 studies (Nepal and Mexico) reported positive and significant effects of CCT on C-section at the last childbirth among beneficiaries	Findings: - Majority of programmes report increases in skilled or facility-based delivery - Schmidt *et al.* (Bangladesh) analyze the use of C-section and do not find a distortion
PNC	- Appears not to have been incentivized in these programmes	- Most articles do not specify which services were covered by the insurance programme	- PNC exempted in 2/13 programmes	- 2 programmes provided incentives for postpartum check-ups	- All programmes cover PNC
	Findings: - No results reported	Findings: - 3 studies showed a consistently positive relationship between insurance and the use of PNC	Findings: - No effects reported	Findings: - 2 studies found negative but insignificant effect of CCT on postpartum visits, with an overall effect-size of 6% decline in postpartum visits	Findings: - 5 studies of 3 programmes report increases in PNC
Family planning	- Various FP indicators linked to incentives in 5 out of 9 programmes (Cambodia, DRC, Egypt, Haiti, and Rwanda)	- Most articles do not specify which services were covered by the insurance programme	- Unclear if FP part of exempted services; may be included in ANC or PNC counselling, and in PHC services generally in Afghanistan	- Unclear if this was directly incentivized. FP counselling may be included in other visits, such as ANC or children's health check-up visits	- 3 programmes cover FP services
	Findings: - No effect on the number of new and continuing users where that was incentivized (Cambodia, DRC, and Rwanda) - No effect on reduced discontinuation (Haiti) - Increase in availability of FP commodities (Haiti) - Quality of family planning counselling and service provision improved in Egypt	Findings: - No studies reported on the use of FP services	Findings: No effects reported	Findings: - 1 programme reported on contraceptive-use, finding that beneficiaries were 16 percentage points more likely to use a modern contraceptive method than non-beneficiaries	Findings: - 1 study reported increases in FP-use (India)

The strongest results are for labour and delivery: the majority of studies that report on skilled birth attendance or facility-based deliveries show incentives to providers and consumers correlated with improvements in these indicators. Similarly, among studies that report on the effect of incentives on caesarean sections, the evidence shows incentives correlated with increased use of caesarean section. Where this service was not directly incentivized, such as in ‘broad’ CCT programmes, the reason for the increase is unclear but may be due to incentives in payment mechanisms.

The evidence around antenatal care (ANC) is also mostly positive, with ANC visits increasing across programmes, although there are exceptions, including some where other health benefits were observed. For example, a rigorous impact evaluation of PBI in Rwanda showed no increase in the quantity of ANC visits but an increase was reported in the quality of ANC as measured by provision of tetanus toxoid vaccine. The authors attribute this to the relatively modest payment to the providers for ANC visits, which may not have been enough to encourage providers to exert the effort to get women to come back for those visits. However, once women were at the facility, tetanus toxoid could be administered without significant extra effort (9,10).

Incentives for postnatal care (PNC) and family planning (FP) were less common across programmes, and, overall, the evidence is weak. Among the insurance and voucher studies that reported results for PNC, there was a consistently positive relationship between the incentives and the use of postnatal care. No effect on PNC was reported in supply-side or user fee exemption programmes, and the two studies that measured the effect of CCT programmes on PNC found negative but insignificant results (11,12). Only one CCT programme—Mexico's *Oportunidades*—reported on contraceptive-use, with finding that beneficiaries were 16 percentage points more likely to use a modern contraceptive method than non-beneficiaries (13). Various FP indicators were linked to incentives in 5 out of 9 supply-side programmes (Cambodia, Democratic Republic of Congo, Egypt, Haiti, and Rwanda). The overall effect was weak; however, voucher, insurance, and user fee studies reviewed here did not report on FP.

#### Quality of care

Quality of care is crucial for better health and has many dimensions, including structural quality, clinical quality, and patients’ satisfaction (14). Improved quality of care can be supported through incentive approaches in a variety of ways (15). Providers’ participation in an incentive scheme may be made conditional on reaching a minimum threshold of quality, such as accreditation. In supply-side programmes, payment to providers can be linked to adherence to clinical guidelines, such as content of care indicators or can be conditional on a score on a quality checklist, index, or patients’ satisfaction survey.

Quality may also be enhanced indirectly. Programmes that provide incentives for increases in service utilization may motivate providers to improve quality to attract patients. Greater revenue from incentives can also be reinvested in facilities to improve quality.

Few studies were explicit about whether quality was incentivized in the programmes they evaluated, and few reported on quality effects. Among studies that do report on quality, the evidence is mixed. Some studies report improvements in quality as measured by various contents of care indicators, which are, in some cases, directly incentivized and, in some cases, not. For example, a study in Bangladesh found that almost all facilities that achieved quantity targets around facility-based delivery also saw improvements in the quality of deliveries as measured by the use of a partograph and readiness of the labour ward (16). Similarly, a study of Mexico's CCT programme reports a positive correlation between incentive and the number of MOH-recommended prenatal procedures provided during antenatal visits as well as the number of iron supplements provided (17).

Very few of the demand-side incentive studies discuss mechanisms of payment to providers, which can have a significant impact on the quality of service provision. Although the voucher programmes pay providers fees for services delivered, none of the programmes reviewed conditioned payments on quality. Although all voucher programmes report engaging accredited facilities, the content of accreditation is not reported, and improvements in accreditation scores over time are not detailed.

Understanding mechanisms of payment to providers is important since increasing demand without commensurate supply-side support may actually damage quality. For example, one user fee study from South Africa showed a decrease in ANC service-use when fees for curative care were removed. The authors attribute this decrease in preventive care-use to increased congestion in clinics and reduced consultation times (18). The evidence on the impact of user fee exemptions on quality suggests that policy-makers should exercise caution, given that fee exemption policies may directly reduce facility revenues. Averting negative supply-side effects relates to “whether policies were effectively put into place to ensure that facility operating budgets and provider incomes did not decrease, as well as the pre-existing infrastructure, human resources, and supply chain systems in place prior to the policy change” (3).

#### Outcomes

The evidence demonstrating impact on health outcomes is weak across all studies and all incentive instruments because few studies were powered or designed to establish such causal links.

An evaluation of *Oportunidades* reports an 11% decline in maternal mortality in regions where at least one locality was participating in the programme and shows a decline in the incidence of low birthweight (the proportion of infants born with low birthweight declined by 4.6%) (19). The evaluation of India's *Janani Suraksha Yojana* (JSY) programme reports large declines in perinatal and neonatal deaths but findings for maternal death were non-significant (20). Three of the insurance studies examined the effect of insurance on maternal mortality but only one from China was rigorously conducted. The study found no effect of insurance enrollment on pregnancy-related deaths (which are already low) (21).

Overall, the evidence on health outcomes is inconclusive, partly due to the small number of studies focusing on outcomes, the weakness of some evaluation designs, and conflicting findings among the studies.

#### Equity

The effects of financial incentives on equity are not well-documented as few studies have examined effects across wealth or income subgroups. Available evidence is mixed.

Most of the demand-side incentive programmes target poor populations. Voucher and CCT programmes typically target low-income women, either through means-testing, geographic targeting, or a mix of both. The public health insurance and private micro-insurance programmes (such as community-based health insurance) also typically target low-income individuals excluded from formal-sector schemes. Most user fee exemption and PBI programmes do not explicitly target individuals according to economic status, except the programmes and policies implemented in regions where most people are poor [One user fee study (Ethiopia) noted that, while outpatient-level service fees were exempted for everyone, a waiver system for the poorest existed for hospital-level services, including obstetric surgeries. So, at least one country did target hospital-level waivers to the poor].

A recent study of removal of user fees for caesarean section in Mali found that wealthier women were obtaining a significantly greater share of free caesarean sections than poor women—a finding they attribute to persistent geographical, transportation, and cultural barriers to seeking and accessing facility-based care (22). In India, an unpublished evaluation of JSY by Mazumdar, Mills, and Powell-Jackson reports that the programme was more effective for less-educated, poor and ethnically-marginalized women (23).

Insofar as the poorest are the farthest removed from healthcare facilities, the insurance studies provide conflicting evidence regarding whether health insurance can overcome geographic barriers to care. In the DRC, there was no difference in the rate of caesarean sections among the insured population, regardless of individuals’ residential distance to facility; in contrast, the rate of caesarean sections was lower among uninsured individuals who lived further from the facility (24); a study in India found that, as distance from the hospital increased, utilization of hospital services decreased, regardless of insurance status (25).

Voucher schemes in Bangladesh and Pakistan show that vouchers increased service utilization more among the poor than the non-poor, and early results from an ongoing evaluation of five voucher schemes in Bangladesh, Cambodia, Kenya, Uganda, and Tanzania also show positive results on service utilization and equity (5).

## DISCUSSION

This summary of the reviews suggests that various types of financial incentives can increase service utilization and, in some cases, improve the quality of maternal and neonatal health services across a variety of geographic, political and social contexts. In this section, we discuss questions and issues raised by the review.

### Context matters but how much?

Certain incentive models tend to be found in certain regions. For example, broad CCT programmes are found almost exclusively in Latin America while narrow CCTs group mostly in Asia. The majority of voucher programmes identified in our reviews are located in Asia, with only a sprinkling in sub-Saharan Africa (SSA); supply-side programmes dominate in Africa and Asia. This grouping is probably partly due to the fact that countries learn from others in their region. A positive experience with CCTs in Mexico spurred other countries in Latin America to test the approach, much as a positive experience in Rwanda spurred other SSA countries to test PBI.

This geographical grouping also raises the question of whether certain strategies are more appropriate in certain contexts. Certainly, efforts to increase demand are most appropriate where the supply is simultaneously being strengthened; approaches that tackle both supply and demand may be more effective. A study in Bangladesh that compared providing incentives to providers with a combination model of supply-side incentives plus cash transfers to women for delivering in a facility found that the combined incentive model had a larger effect on the numbers of institutional deliveries than performance incentives to providers only (16). More countries, such as Afghanistan, Malawi, Rwanda, and Senegal, are beginning to incorporate rewards for patients as complements to their supply-side programmes in recognition that improvements at the facility level alone are rarely enough to overcome barriers that the families face when deciding to seek care.

Another central question is whether, or the degree to which, financial incentive schemes require certain conditions to flourish. The context within which any programme is implemented can have a profound impact on whether it achieves its objectives. Geographical factors, such as ruggedness of the terrain or remoteness of health facilities and communities, can affect access to care, availability of essential supplies, and motivation of health workers. Political and economic conditions and events may affect macro-economic stability and whether there are adequate numbers of skilled providers, strong health management and information systems, and functioning supply chains.

While the review by Glassman *et al*. suggests that contextual factors underpin the effectiveness of CCTs (8), other reviews suggest that even in unstable and disrupted environments (e.g. Afghanistan, DRC, and Haiti, among others), incentive programmes can have an impact on the use of maternal health service and, in some cases, quality of care and can strengthen health systems in the process. For example, the need to generate timely and reliable data on which to base payment may strengthen health information systems, particularly in supply-side schemes that rely on routine service-delivery data.

### Perverse incentives, distortions, and unintended consequences

In any incentive programme, there is the potential for unintended consequences. On both demand and supply sides, there is the risk that incentives will encourage false reporting, cheating, or other forms of fraud. The stronger the incentive to providers to simply increase the quantity of services, the more likely benefits will accrue first to those who are easiest to reach, i.e. individuals who are usually better-off socially and economically than others, which may exacerbate inequities. Incentives to providers to increase quantity can also result in the provision of unnecessary services or providers pressuring patients to accept services they do not need or desire.

Moreover, if programmes focus on increasing demand without providing commensurate support to the supply side and providers face burgeoning demand together with shortages of essential inputs, like drugs and supplies, quality of care may suffer. For example, a study from Mauritania measured quality based on whether partographs were correctly filled in based on a review of delivery-records at facilities covered by insurance; this study found a decrease, over time, in the percentage of deliveries with a partograph filled in—something the authors hypothesized was due to the increased workload faced by service providers as a result of an influx of insured patients while providers’ pay remained unchanged (26). Some user fee studies note that the loss of revenue from user fees in some cases led to stock-outs of drugs and supplies, negatively affecting the quality of care provided and resulting in some facilities reinstituting fees.

On the demand side, concerns that providing per-child benefits from birth (in the case of broad CCTs) or incentives for delivery could stimulate increases in fertility are largely unsubstantiated. Only CCT studies from Honduras, Nicaragua, Mexico, and Uruguay report impact on age-specific and total fertility rates (8). The overall effect is negligible, with a 0.2% increase and range from a 4% increase in Honduras to a 1% decrease in Nicaragua. The Honduran programme provided women with per-child benefits from birth, a programme design that may have resulted in this change. Meanwhile, a CCT evaluation in Pakistan found that a beneficiary's probability of giving birth was 8 percentage points less than a non-beneficiary; the beneficiaries were more likely to have a smaller number of children and more likely to be older at marriage (27).

Avoiding distortions depends, in large part, on how programmes are designed and the rigor with which programmes are monitored. In terms of design, incentivizing only one service or a handful of services at much higher rates than others may cause distortions. Exempting fees for caesarean sections but not normal deliveries in Mali raised concerns about exactly this (22). The best way to avoid distortions is to ensure that incentives paid for certain indicators are not significantly higher (or lower) than those paid for other indicators. Subsidizing a package of maternal health services may also be preferable. There are also many approaches that can help direct benefits toward the poor: eligibility can be limited to poor people or families or deprived geographic areas; and rewards to providers can be higher for those serving disadvantaged populations.

Moreover, although most incentive programmes (and evaluations) have focused most squarely on increasing and measuring quantity, tackling quality of care is urgently needed. Efforts should be redoubled to incentivize quality care by, for example, conditioning payment to providers on quality, not only in PBI programmes but also in insurance and voucher schemes. Combination approaches should also be increasingly tried and evaluated so that demand is spurred and quality improved in tandem. Finally, facilities should be supported with the necessary equipment, supplies, supervision, and training, to provide the services required when demand increases.

### Strengthening evidence

Our review shows overall positive results in key areas; however, as already mentioned, there is significant variation in study designs. Studies that use randomization establish most robustly the causal impact of the incentives on results. Econometric methods can control for most, but not all, potential confounding factors and various types of selection bias. Very few studies used randomized approaches and a subset relied on econometric techniques. Thus, aside from the literature on CCTs, many study designs were not strong enough to conclusively disentangle the effect of the incentives from other confounding factors or secular trends. Furthermore, most studies were of short duration, meaning that few studies could evaluate the long-term effects of incentive programmes.

Moreover, comparing results across countries and the type of incentive is a challenge, in part, because performance indicators are not consistent across studies, and internationally agreed-upon indicators for measuring quality in MNH are still being developed. As noted above, evaluations of demand-side initiatives typically did not examine supply-side effects, such as workload, payment to and satisfaction of the providers, or service quality.

Although randomized control trials (RCTs) are often considered the gold standard of evaluation, the challenges around implementing RCTs are well-documented (28-32). Finding a ‘pure’ control area can be difficult. There are also often political barriers to randomization: governments may have interests in assigning where a programme is implemented and whether it is piloted, and the interests of researchers can be incompatible with political goals. RCTs are also expensive and require holding the environment and programme constant; the former can be challenging in development landscapes with myriads of simultaneous interventions; the latter is not necessarily desirable since learning from implementation and revising as you go are important elements of success.

In complex and ever-changing systems, measuring the effect of programmes that aim to change systems and behaviour requires a mixed-methods approach. To understand whether and in what contexts incentive approaches contribute to better MNH service-use, quality, and outcomes, it is important to employ strong methods from all disciplines, including both qualitative and quantitative approaches. Qualitative research and process documentation are particularly important for capturing lessons of design and implementation, knowledge which often goes unpublished but is of critical interest to governments, evaluators, practitioners, donors, and the global health community, both as a means to improve and revise programmes and to inform policy (32,33). Efforts should be made to capture these kinds of lessons from practitioners and to share the knowledge with other stakeholders.

### Sustainability

Questions about sustainability are ever-present in the field of development, and incentive programmes are no exception. Whether financial support for incentive programmes is sustained depends on many factors, such as political support, perceptions of impact, country ownership, participation and leadership, and integration into country-specific structures and systems. Cost-effect-iveness is also a key; yet, there is little evidence of cost-effectiveness available for any of the incentive instruments. Going forward, cost-effectiveness data should be reported, not only for the interventions themselves but for a standard outcome, such as disability-adjusted life years (DALYs) so that interventions can be compared [The DALY is a measure that captures the loss of healthy years, either in terms of quality (due to ill-health or disability) or in terms of quantity (due to early death). The DALY was initially developed to provide a picture of the global burden of disease, and it is increasingly used as a way to measure the health impact of health projects. As a common unit, the DALY enables comparisons of the effectiveness and cost-effectiveness of various projects]. More research is needed to answer the question whether incentive instruments are cost-effective and whether there are alternative (and cheaper) ways to get similar results.

Another question is whether and how long improved behaviour relies on the existence of the financial incentive. Will patients’ behaviour, for example, revert when user fees are re-introduced, or vouchers or CCTs become unavailable? Will providers’ behaviour return to the status quo if payment reverts to a non-performance-based mechanism? There is no evidence from LMICs to show that removing financial incentives damages the intrinsic motivation of patients or providers, or even to show that things return to the status quo. However, some evidence suggests that there can be a learning effect whereby women with longer exposure to incentives make greater use of services, even those not directly incentivized, perhaps because the programme has increased their appreciation for such services. For example, an evaluation reviewed by Glassman *et al*. in this series found an increase in the last delivery attended by a physician/nurse versus a traditional midwife in Mexico, although the CCT only specifies the use of adequate antenatal care, not facility-based delivery, or the use of a skilled birth attendant (34). Authors are also aware of anecdotal evidence from voucher programmes: in a voucher programme introduced in Kenya in 2006, uptake of the family planning voucher fell far short of anticipated levels in the first several years but increased considerably in 2010, nearly tripling from what was observed in phase one. The increase was probably driven by a combination of things, including provision of education on the benefits of FP to providers and community education and marketing. More robust research is needed to explore the long-term effects of introducing and removing financial incentives on behaviour.

Based on insights gleaned from the array of evidence reviewed in this series, we offer below some suggestive ideas to practitioners, programme managers, and policy-makers for strengthening financial incentive programmes and research. Much has already been written about strengthening incentive programmes. The Health Results Innovation Trust Fund of the World Bank provides a variety of tools and case studies; USAID's Health Systems 20/20 Project also provides cases and practical tools for practitioners; and the Performance-based Financing Community of Practice regularly shares experiences, lessons, and challenges from the field. It is to and within this lively space, therefore, that we share our insights and suggestions for strengthening research, policy, and practice.

### Suggestions for strengthening financial incentive programmes

1. *Mitigate the risks*: Programmes should be designed with a view to mitigating the risks of distortions and perverse incentives by:
  - choosing the amounts for payment and indicators/services to be incentivized carefully;
  - ensuring that incentives paid for certain indicators/services are not significantly higher or lower than those paid for other indicators; and
  - establishing the independent verification systems, with sanctions for misreporting.
2. *Embrace adaptation*: Context matters, and indicators should reflect context-specific maternal health needs. Other design features, from amounts and frequency of payment to demand-side subsidies, should be tailored to local realities.
3. *Take an interdisciplinary approach*: The design of incentive programmes can benefit from multidisciplinary expertise from economists, clinicians, public health practitioners, health system experts, and policy-makers.
4. *Strengthen incentives for quality and equity*. Programmes can be designed from the start to enhance equity and promote quality. Rewards for increases in the quantity of services should be conditioned on quality in payment schemes for providers, and efforts should be made to better target benefits to the poorest and the hardest-to-reach population (15, 35). This can be done in a variety of ways, such as by limiting eligibility to poor people or deprived regions and by paying providers more for serving in disadvantaged areas.
5. *Consider combining demand- and supply-side approaches*: Combining supply and demand approaches may help ensure that increases in demand are directed at high-quality service provision and that the provision of services can match increased demand while conversely tackling the community-based barriers to ensuring maternal health (cultural norms, transportation costs, etc.), which are often beyond the scope of facilities to tackle.

### Suggestions for strengthening research

1. *Stronger study designs*: Researchers should strive to ensure plausible comparison groups or health indexes in study designs, enabling assessment of the counterfactual (what would happen in the absence of the financial incentive) at the individual or group level wherever possible. Funders of incentive programmes should invest in independent evaluation.
2. *Longer time horizons*: There is a need for evaluations of more mature incentive programmes to identify longer-term effects on maternal healthcare utilization, outcomes, and service quality. Early evaluations are important but give an incomplete picture, especially as both providers’ and consumers’ behaviours may adapt to the policy over time, and initial effects may not persist.
3. *Implementation research and qualitative methods*: More priority should be given to complementing impact evaluations with broader strategies that also capture lessons about design and implementation, using qualitative research methods and approaches. Such studies can help understand the ‘how’ and ‘why's of changes observed from quantitative evaluations. Programmes themselves should strive to have robust reporting and verification systems, capable of detecting unintended consequences and should develop systems to document the implementation process, and such information should be shared across communities of practices and other platforms. Incentives are powerful, and incentive programmes should not be static: learning and the flexibility to revise are the key.
4. *Study a broader range of issues, including the following*:
  - *Equity and targeting:* There is a need to measure the impact of incentives on equity of access to MNH care and of distribution of healthcare resources across socioeconomic groups, between rural and urban women, and for marginalized groups The question of how best to target exemptions to priority subgroups also needs continued study.
  - *Supply-side effects:* It is critical for programmes to consider ‘demand side’ to evaluate the effect of increasing demand on providers. More attention should also be paid to the supply-side components of insurance, voucher, and user fee schemes, and evaluations should examine the effect on the quality of various provider payment approaches.
  - *Cost-effectiveness of different incentive approaches*: The cost-effectiveness of incentive approaches compared both to each other and to non-financial approaches to stimulating access and quality should be studied.
  - *Variables that affect the impact of incentives*: The variables that affect the impact of incentives, such as whether they are applied in government or private settings, deserve greater scrutiny.
  - *Unintended consequences, perverse effects*: Stigma, family pressure to use services, supervisors’ pressure to falsify data, and other effects may result in a decrease in women's choices, promote inappropriate care or undeserved payments, and otherwise cause undesirable consequences. Attention should be paid to detecting unintended consequences and revising programme design accordingly. Research should also aim to learn about positive unintended consequences or spillover effects as these may also hold valuable lessons for future programmes.
5. *Development of quality of care indicators*: Quality measures for maternal health services could benefit from standardization to enable assessment of causal pathways. Such indicators are likely to improve health outcomes as well.

### Conclusions

Reviews of the Evidence Summit show that financial incentives can enhance demand for and improve the supply of maternal health services—a finding that is true across instruments and geographic locations. Some programmes also show improvements in quality of care. Evidence on the impact on health outcomes and equity is weak, and few evaluations describe details of design and implementation. Moreover, in many studies, it is difficult to isolate the incentive effect from the many other potential confounding factors.

On the whole, however, the evidence suggests that financial incentives can enhance utilization of maternal healthcare services, quality and equity, if programmes are carefully designed and implemented. More robust impact evaluations are needed, complemented with qualitative studies to understand how stakeholders respond to incentives and the processes that lead to impact as well as to identify problems and corrective actions to improve project implementation. A more comprehensive and consistent methodology for measuring the quality of MH services would also help ensure that studies capture meaningful (and comparable) measures of quality.

For decades, governments and health systems have tried various approaches to enable women to utilize health services that facilitate healthy pregnancy and delivery outcomes. The Evidence Reviews strongly suggest that a range of supply- and demand-side financial incentives can enhance utilization and quality of maternal health services. Improving the evidence to inform appropriate use of financial incentives for maternal and neonatal health requires more methodologically-robust studies designed to assess attribution, sustainability, cost, equity, and outcomes.

## ACKNOWLEDGEMENTS

The views and opinions expressed in this paper are those of the authors and not necessarily of the United States Agency for International Development (USAID), the United States National Institute of Allergy and Infectious Diseases (NIAID), or any of the affiliations of the authors. Among the authors, ESH is paid by the National Institute of Allergy and Infectious Diseases and was on assignment with the Bureau for Global Health at USAID during the planning and execution of the Evidence Summit. RLB is paid by Virginia Commonwealth University in Richmond, VA, USA. During the planning of the Evidence Summit, he was a Jefferson Science Fellow assigned to the USAID and received supplemental support from the National Academy of Sciences. Currently, he receives part of his salary through an Intergovernmental Personnel Act arrangement with USAID. Lindsay Morgan was funded through the Maternal and Child Health Integrated Program of USAID for this work. At the time of the Summit, Marge Koblinsky was funded by the Maternal and Child Health Integrated Program through John Snow, Inc.; she is now funded by Johns Hopkins Bloomberg School of Public Health to work with USAID.
